# Cost-effectiveness analysis of initial nonoperative management versus emergency laparoscopic appendectomy for acute complicated appendicitis

**DOI:** 10.1186/s12913-020-05839-6

**Published:** 2020-11-09

**Authors:** Kiyoaki Sugiura, Keiichi Suzuki, Tomoshige Umeyama, Kenshi Omagari, Takeo Hashimoto, Akihiko Tamura

**Affiliations:** Department of Surgery, National Hospital Organization Tochigi Medical Center, 1-10-37, Nakatomatsuri, Utsunomiya, Tochigi, 320-8580 Japan

**Keywords:** Cost-effectiveness, Markov model, Laparoscopic appendectomy, Complicated appendicitis, Japan

## Abstract

**Background:**

The evidence regarding the safety and efficacy of nonoperative management is growing. However, the best treatment strategy for acute complicated appendicitis remains controversial. We aimed to evaluate the cost-effectiveness of treatment strategies for complicated appendicitis patients. This study sought to determine the most cost-effective strategy from the health care-payer’s perspective.

**Methods:**

The primary outcome was an incremental cost effectiveness ratio (ICER) using nonoperative management with or without interval laparoscopic appendectomy (ILA) as the intervention compared with operative management with emergency laparoscopic appendectomy (ELA) alone as the control. Model variables were abstracted from a literature review, and from data obtained from the hospital records of Tochigi Medical Center. Cost-effectiveness was evaluated using an ICER. We constructed a Markov model to compare treatment strategies for complicated appendicitis in otherwise-healthy adults, over a time horizon of a single year. Uncertainty surrounding model parameters was assessed via one-way- and probabilistic-sensitivity analyses. Threshold analysis was performed using the willingness-to-pay threshold set at the World Health Organization’s criterion of $107,690.

**Results:**

Three meta-analysis were included in our analysis. Operative management cost $6075 per patient. Nonoperative management with interval laparoscopic appendectomy (ILA) cost $984 more than operative management and produced only 0.005 more QALYs, resulting in an ICER of $182,587. Nonoperative management without ILA cost $235 more than operative management, and also yielded only 0.005 additional QALYs resulting in an ICER of $45,123 per QALY. Probabilistic sensitivity analysis with 1000 draws resulted in average ICER of $172,992 in nonoperative management with ILA and $462,843 in Nonoperative management without ILA. The threshold analysis demonstrated that regardless of willingness-to-pay, nonoperative management without ILA would not be most cost-effective strategy.

**Conclusions:**

Nonoperative management with ILA and Nonoperative management without ILA were not cost-effective strategies compared with operative management to treat complicated appendicitis. Based on our findings, operative management remains the standard of care and nonoperative management would be reconsidered as a treatment option in complicated appendicitis from economic perspective.

## Background

Appendicitis is one of the most common acute abdominal diseases and emergency surgeries [1]. Appendectomy is the treatment of choice, and laparoscopic appendectomy has become more common [2] [3]. However, management of patients whose appendicitis is complicated by perforation, cellulitis, or abscess remains controversial.

Patients with complicated appendicitis undergoing immediate surgery might require larger colonic resection and have higher complication risk and longer hospital stay [4] [5] [6]. Therefore, these patients can be treated with antibiotics with image-guided drainage, as needed, without surgery, in the acute setting. This initial nonoperative management is safe, and planning an interval appendectomy in patients with complicated appendicitis appears successful [7] [8]. However, it is also questionable whether such conservative measures should be followed by elective interval appendectomy. A meta-analysis of 61 studies concluded that interval appendectomy may not be necessary in patients who respond to nonoperative management because the pooled risk of recurrent appendicitis was < 10%, and the incidence of malignancy was < 2% [9].

Therefore, in Japan, there is ongoing debate over the management of complicated appendicitis, and clinicians continue to use both operative and nonoperative treatment strategies. For comparison of competing management strategies in the setting of clinical complexity, the cost-effectiveness analysis is particularly useful to assess which treatment strategy is more effective relation to its cost. To date, and to our knowledge, no study has examined the cost effectiveness of treatment strategies in patients with complicated appendicitis in Japan. Using the technique, we performed a cost-effectiveness analysis of operative management with emergency laparoscopic appendectomy (ELA) alone as the first-line therapy in patients with complicated appendicitis in a municipal hospital in Japan.

## Methods

### Reference case

For our analysis, the reference case was an adult diagnosed with complicated appendicitis with confirmatory abdominal imaging. The patient was > 18 years of age without comorbidities that would substantially increase their risk of complications from laparoscopic appendectomy and image-guided drainage. Complicated appendicitis was defined as appendiceal inflammation with the presence of appendiceal abscess, cellulitis, or extraluminal air on initial abdominal computed tomographic images.

### Treatment strategies

We compared the cost-effectiveness of the three following treatment strategies from a health-care payer’s perspective in Japan (health insurers, and the government): (1) operative management with elective ELA, (2) initial nonoperative management with interval laparoscopic appendectomy (ILA) at 2 months, and (3) nonoperative management without ILA. Nonoperative management entailed hospitalization for 5 days with intravenous cefmetazole as a 3-day course of antibiotics. Computed tomography-guided percutaneous abscess drainage was performed if necessary. All nonoperative treatment failures regardless of the specific indication (e.g., failure to improve, worsening vital signs or laboratory parameters, provider or patient preference) required delayed laparoscopic appendectomy in the same hospitalization. Failures occurring after discharge were considered recurrent appendicitis, and patients with recurrent appendicitis underwent ELA.

### Decision model

**Fig. 1:**
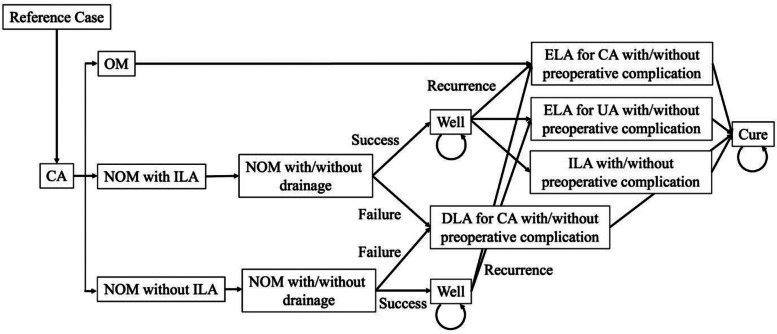
Treatment strategies for complicated appendicitis. Abbreviations: CA complicated appendicitis, NOM nonoperative management, UA uncomplicated appendicitis, OM operative management, ELA emergency laparoscopic appendectomy, ILA interval laparoscopic appendectomy, DLA delayed laparoscopic appendectomy, RA recurrent appendicitis

For decision analytical modeling, we used a Markov model to simulate costs, health outcomes, and cost savings while comparing the three treatment strategies (Fig. 1). Given that patients’ health states generally return to baseline within 1 year after acute appendicitis, we chose to condense our model by applying the total long-term risk of recurrent appendicitis after nonoperative management into a single year [10]. therefore, we did not include background mortality in the simulation. Each cycle was defined as 1 month in length. The decision model was constructed and analyzed using R, version 3.5.0 with heemod package(R Foundation for Statistical Computing, Vienna, Austria).

#### Probabilities

Probabilities of clinical events were abstracted from a literature review (Table 1) [4] [11] [12]. The literature review was performed using the PubMed database, using the terms, “Acute appendicitis,” “Nonoperative,” “Conservative,” “Nonsurgical,” “Appendectomy,” “Complicated,” “Abscess,” “Perforated,“ and “Phlegmon.” These terms and their combinations were also searched as text words. The search was performed on November 2018, and English language restriction was applied. Because there was limited evidence of the efficacy of laparoscopic appendectomy compared with nonoperative management in treating complicated appendicitis, the inclusion criteria were randomized clinical trials and meta-analyses comparing nonoperative management and operative management (which includes laparoscopic appendectomy) in complicated appendicitis. Exclusion criteria were narrative reviews, studies without control groups, case reports, case series studies, and studies involving pediatric patients.

**Table 1:**
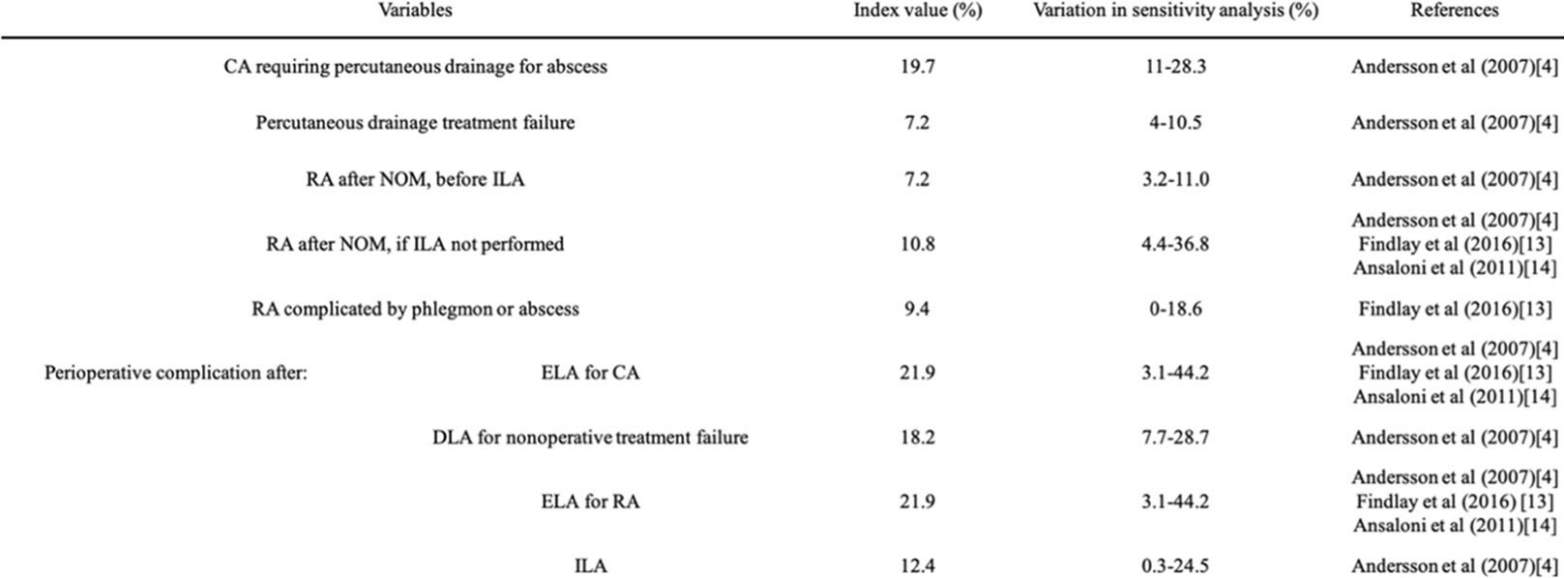
Model variables: probabilities.

### Costs

Costs were estimated from a health-care payer’s perspective; therefore, only direct medical costs were included. The cost of laparoscopic appendectomy is an unexplored field. Thus, careful attention was paid to the costing methodology because there is no gold standard. Therefore, for cost analysis, we used a micro-costing method in which the actual monetary health care costs are categorized within the main category: Diagnostic procedures, drugs, ward care, and operating room cost [13] [14]. Data for health care costs were based on the diagnosis-procedure combination/per-diem payment system and fee-for-service, and specific material expenses between 01 April 2011 and 31 March 2018 were retrospectively obtained from the electronic database of the Tochigi Medical Center (Table 2). Costs of perioperative complications were estimated by the increase in average hospitalization cost for complicated appendicitis with complication or comorbidity based on data from our hospital records. We assumed that the costs for outpatient follow-up after hospitalization were equivalent between treatment groups, so these costs were not included in this analysis. Costs are expressed based on US dollars, 2018.

**Table 2:**
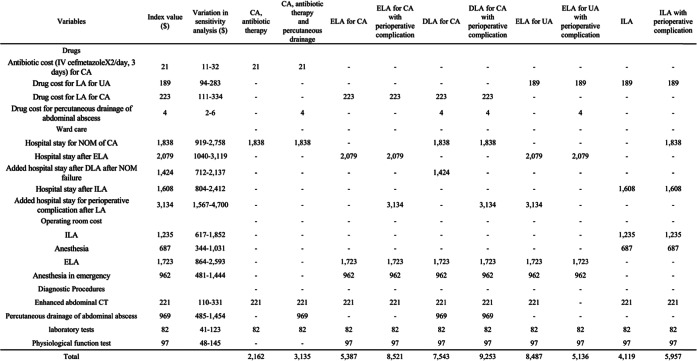
Model variables:Total direct Medical costs.

### Health-related utility

The primary measures of effectiveness in the present analysis were quality- adjusted life years (QALY) gained. To estimate total QALYs in the Markov model, QALYs were calculated by multiplying the health care-related quality of life (HRQoL) score of a disease state by the duration of time a patient spent in that disease state. We obtained the HRQoL factors from our literature review, and data are shown in Table 3 [10] [15] [16] [17]. Because there were few quality-of-life estimates in the literature for the health states of appendicitis [18] [19] [20]. we used the method proposed by Wu et al., in which the utility of undergoing various treatments for appendicitis is estimated by multiplying established utilities by the average duration of hospitalization and recovery from complicated appendicitis associated with each therapeutic strategy [15] [16] [17]. Additionally, we utilized 1-time decreases in QALY for unplanned emergency room visit with readmission and percutaneous drainage of abdominal abscess [17]. For example, given that the utility of patients hospitalized for nonsurgical treatment was assumed to be reduced to 0.98 for the duration of the hospitalization and the mean length of stay for these patients was assumed to be 5 days, and One-time QALY reduction for unplanned emergency visit was subtracted from total QALYs, the utility for that a month cycle (28 d) was calculated as ([0.98*5] + [23*1])/28–0.005 = 0.991 (for patients with antibiotic therapy).

**Table 3:**
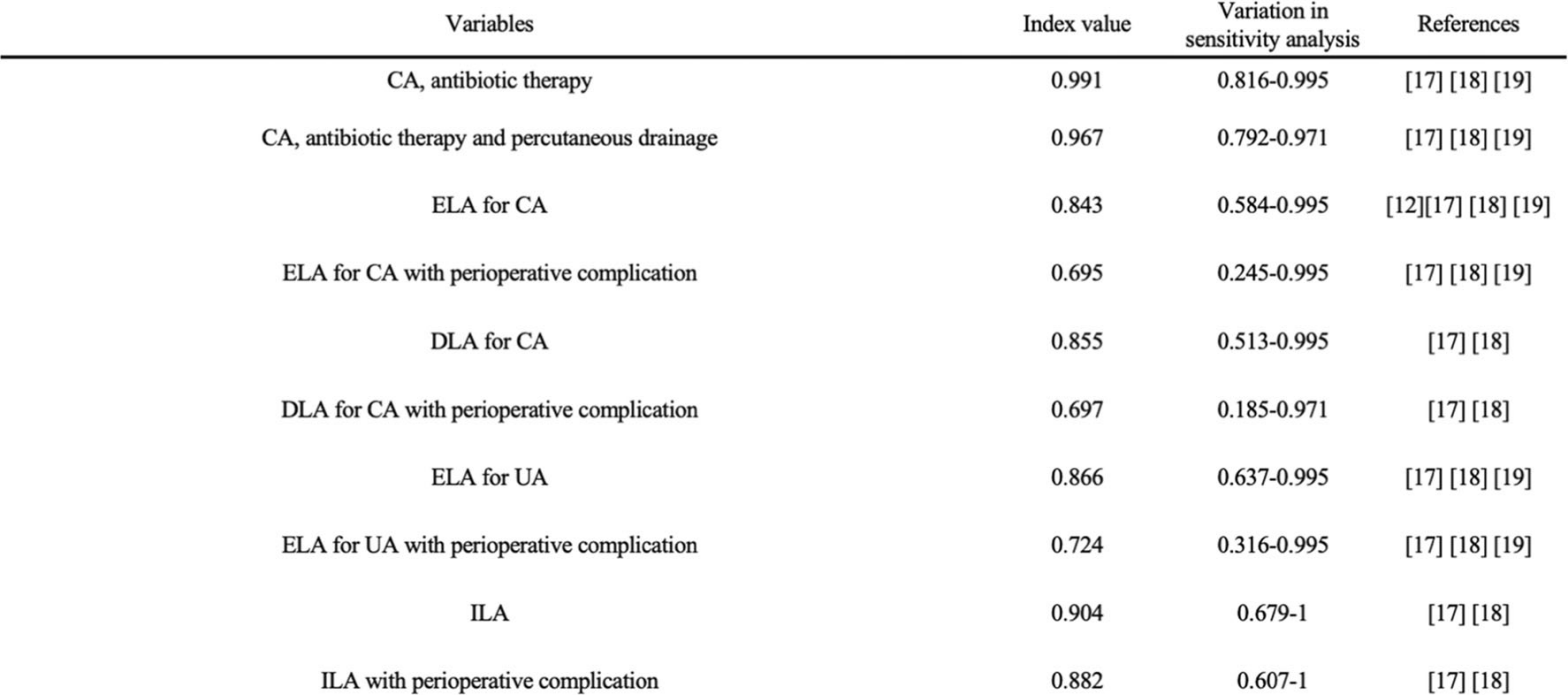
Model variables: health-related quality-of-life factors.

### Cost-effectiveness analysis

Cost-effectiveness was evaluated using the incremental cost-effectiveness ratio (ICER). In this analysis, we defined the willingness-to-pay (WTP) threshold based on the criterion of the World Health Organization that states that an intervention is considered cost-effective if the ICER for QALY is 1–3-fold the gross domestic product per capita [21]. In this analysis, based on the per capita GDP of Japan in 2016 ($37,960), the threshold of $113,880 per QALY for cost effectiveness used in this study. Based on this standard, we defined the cost-effective strategy as the strategy that produced the greater utility without exceeding a threshold of $113,880 per QALY and the very cost-effective strategy as the strategy that produced greater utility without exceeding a threshold of $37,960. Because there is no disease-specific threshold for appendicitis, we provide only GDP-based threshold. We also cited operative management, the current standard of care, as a benchmark intervention for all comparisons. Using operative management as a threshold for acceptable cost-effectiveness, a strategy is considered cost-effective if it is both less costly and more effective than operative management.

### Sensitivity analysis

We performed several sensitivity analyses to evaluate the uncertainty and robustness of the model. For these sensitivity analyses, we selected the parameters that covered all potential areas of uncertainty, such as the probabilities, clinical costs, and health-related utility values. One-way sensitivity analyses assessed the effects of varying key model parameters on the ICER. The variation ranges were established based on the analyzed studies. For costs, we allowed values to vary by ±50% of the index value; variations in sensitivity analysis results are listed in Tables 1, 2 and 3. We also performed a probabilistic sensitivity analysis to assess the impact of sensitivity on the model parameters using a Monte Carlo simulation with 1000 samples. For the probabilistic sensitivity analysis, all model variables (probabilities, costs, utilities) were set as static with triangular frequency distributions. Additionally, a threshold analysis was performed to determine the cost-effective price of each treatment strategy.

### Ethics

This study was approved by the local Ethics Committee on December 3rd, 2018 (number 2018110501).

## Results

### Cost-effectiveness analysis

Compared with operative management, results showed that nonoperative management with ILA cost $984 more and yielded 0.005393 additional QALYs, resulting in an ICER of $182,587 per QALY. Similarly, Nonoperative management without ILA cost $235 more than operative management and yielded 0.00521 additional QALYs, resulting in an ICER of $45,123 per QALY. Table 4 shows the estimated ICER for the cohort.

**Table 4:**
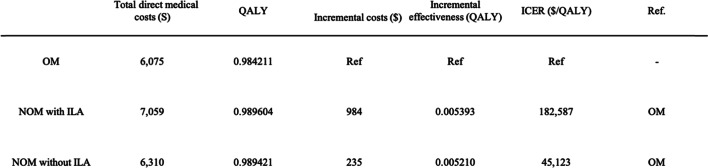
Results of the cost-effectiveness analysis.

### Sensitivity analysis

**Fig. 2:**
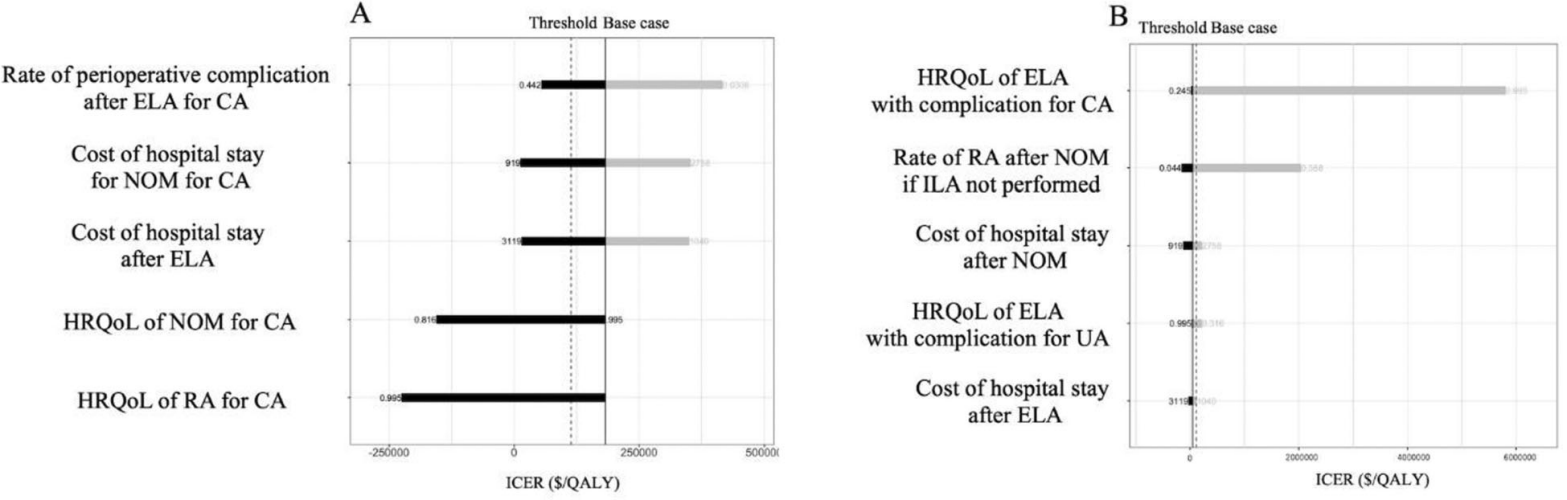
Tornado diagram for one-way sensitivity analysis. a: NOM with ILA, b: NOM without ILA. Abbreviations: CA complicated appendicitis, NOM nonoperative management, UA uncomplicated appendicitis, OM operative management, ELA emergency laparoscopic appendectomy, ILA interval laparoscopic appendectomy, HRQoL health-care related quality of life

The tornado diagram (Fig. 2) graphically and simultaneously displays the one-way sensitivity analysis of some of the parameters. Because of the high number of parameters in our analysis, only key parameters in the ICER threshold are shown in the tornado diagram. In nonoperative management with ILA, the parameter with the greatest influence on ICER was the rate of perioperative complications after ELA for complicated appendicitis. One-way sensitivity analysis revealed that nonoperative management with ILA was the preferred strategy compared with operative management if the rate of perioperative complications after ELA for complicated appendicitis was approximately 20%. In nonoperative management without ILA, HRQoL factors related to ELA with preoperative complications had the greatest influence on ICER. If HRQoL factors related to ELA with preoperative complications improved, nonoperative management without ILA was dominated by operative management. Additionally, the tornado diagram demonstrates a prominent variation in ICER for nonoperative management without ILA. If HRQoL factors related to ELA with preoperative complications increased from 0.245 to 0.995, the ICER of nonoperative management without ILA compared with operative management increased up to $6,000,000.

**Fig. 3:**
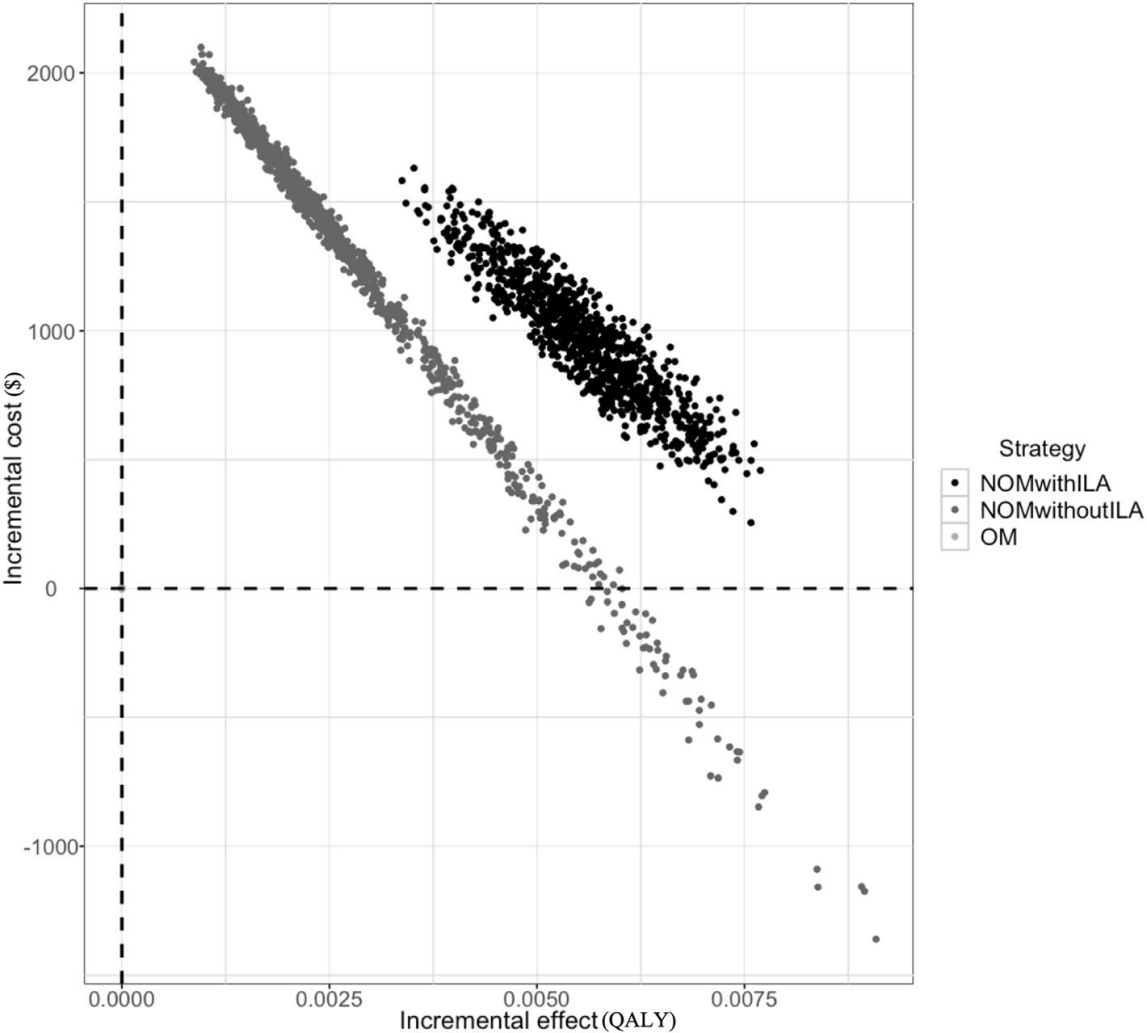
Monte Carlo probabilistic sensitivity analysis. Abbreviations: NOM nonoperative management, OM operative management, ILA interval laparoscopic appendectomy

Monte Carlo probabilistic sensitivity analysis showed average ICERs of $172,992 per QALY for nonoperative management with ILA and $462,843 per QALY for NOM without ILA, both of which were above the threshold of $113,880 (Table 5). Our results demonstrated that nonoperative management with ILA was a consistently more effective and more costly strategy compared with operative management. Consistent with the results of the one-way sensitivity analysis, nonoperative management without ILA showed marked variation in cost and effect. Contrary to the results for the reference case, nonoperative management without ILA was not always the preferred strategy compared with operative management in the 1000 simulated cases (Fig. 3).

**Table 5:**
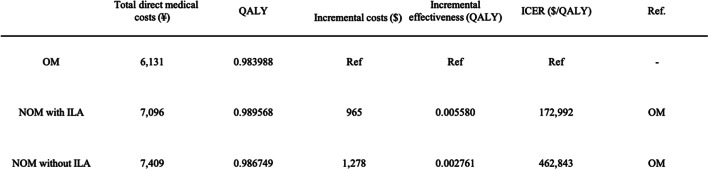
Results of the Monte Carlo probabilistic sensitivity analysis.

**Fig. 4:**
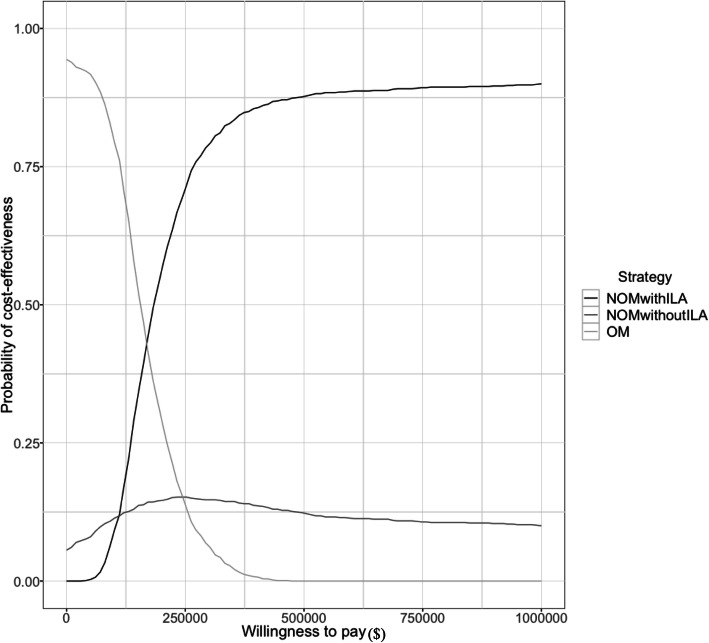
Cost-effectiveness acceptability curve for probabilistic sensitivity analyses. Abbreviations: NOM nonoperative management, OM operative management, ILA interval laparoscopic appendectomy

Uncertainty regarding the cost-effectiveness results also appeared in the cost-effectiveness acceptability curve seen in Fig. 4. The curve shows the probability that nonoperative management with ILA and nonoperative management without ILA would be cost-effective with increasing WTP values. We showed that when the WTP threshold reached its maximum value of $500,000, the probability that nonoperative management with ILA would be more cost-effective than operative management was approximately 80%. In contrast, regardless of WTP, the possibility that nonoperative management without ILA was the most cost-effective strategy was < 20%.

## Discussion

In our analysis, neither nonoperative management with ILA nor nonoperative management without ILA were more cost-effective strategies in the treatment of complicated appendicitis compared with operative management, at the set threshold. Nonoperative management, with and without ILA, provide a minimal incremental benefit at a high ICER compare to operative management. Base case results demonstrated that nonoperative management without ILA was the most cost-effective strategy compared with operative management. However, probabilistic sensitivity analysis showed that nonoperative management without ILA was not a cost-effective strategy among the simulated cases. In contrast, nonoperative management with ILA was the most effective strategy, but also the most costly. Given these findings, we suggest that operative management remains the cost-effective and standard therapeutic strategy, and nonoperative management without ILA and nonoperative management with ILA may not be recommended routinely in the management of complicated appendicitis. To our knowledge, ours is the first study performing an economic analysis comparing the costs of different treatment strategies for complicated appendicitis in the Japanese public health-care system.

Because of continued improvements in the quality and accessibility of computed tomography, the efficacy and feasibility of performing nonoperative management, including targeted intra-abdominal drainage, have increased. In uncomplicated appendicitis, previous studies reported that nonoperative management is successful compared to operative management. Moreover, a recent cost-effective analysis found nonoperative management without interval appendectomy was the most cost-effective strategy for acute uncomplicated appendicitis [17]. However, our study suggested that nonoperative management offers modest benefits at high costs compared to operative management in complicated appendicitis.

In complicated appendicitis, it is important to note that several studies reported higher rates of recurrence after nonsurgical treatment of up to 38% within 1 year [22] [23]. This high chance of recurrence, questioned the feasibility and cost-effectiveness of nonoperative management in our results. There was limited evidence for complicated appendicitis compared to uncomplicated appendicitis. The current study offers a new perspective on management for complicated appendicitis. It is also debatable that whether emergency laparoscopic appendectomy is safe and feasible for complicated appendicitis. It is cautioned that immediate surgical treatment of complicated appendicitis is associated with a more than 3-fold increase in morbidity compared with nonoperative management, and may result in unnecessary ileocecal resection or right-sided hemicolectomy, for technical reasons [4]. However, these data mainly based on open appendectomy. Laparoscopic appendectomy offers superior benefits to open appendectomy, and laparoscopic appendectomy has been used for various types of appendicitis [24] [25] [26]. The results from a previous data with open appendectomy may not be generalized to the modern clinical settings. Given that laparoscopic appendectomy would successfully performed, our study suggested operative management is more cost-effective than interval appendectomy followed by nonoperative management.

Sensitivity analyses in our study indicated that variation in the probability and HRQoL factors for ELA with perioperative complications for complicated appendicitis had a significant influence on outcomes for both nonoperative management with ILA and nonoperative management without ILA. Therefore, we suggest that ELA and its perioperative complications is an important factor in choosing a therapeutic strategy for complicated appendicitis. Randomized control trials have reported postoperative complications following laparoscopic appendectomy for complicated appendicitis, including surgical site infections and intra-abdominal abscess, which are the most common complications following appendectomy [27] [28]. Regarding cost-effectiveness, intra-abdominal abscess leads to a prolonged hospital stay, possible readmission, and the need for subsequent treatment, which increases costs and decreases utility [29]. For preventing intra-abdominal abscess formation, some scholars reported that laparoscopic appendectomy could provide a better visualized abdominal cavity, and a more thorough washout can be performed, perhaps decreasing the incidence of intra-abdominal abscess [27, 30]. However, recent randomized control trials found that irrigation did not decrease the rate of intra-abdominal abscess [29] [31]. The roles of extensive irrigation and routine drainage is still debatable. From the perspective of cost-effectiveness, we speculate that peritoneal irrigation and lavage are considerable procedure in laparoscopic surgery for complicated appendicitis.

It should be noted that no consensus exists regarding the threshold for acceptable cost per QALY ratios in Japan’s national health policy. Therefore, we adopted the World Health Organization’s WTP recommendation for ICER threshold, in our model. This metric is meant to be used solely as a common cognitive anchor rather than as a method of dictating clinical decision-making. Nevertheless, we consider our conclusions in this study robust based on the results of the sensitivity analyses. An acceptability curve showed that the probability of nonoperative management with ILA being the most cost-effective strategy was approximately 50% when WTP was $180,000. Additionally, the possibility of nonoperative management without ILA being the most cost-effective strategy was always < 20% regardless of WTP, demonstrating that both nonoperative management strategies were not cost-effective over a pragmatic range of values for Japanese health care payers. Therefore, operative management remains a standard strategy, and a price reduction would be necessary for nonoperative management strategies to be considered cost-effective.

This study has several limitations. First, because we focused on cost-effectiveness for a relatively short duration, we did not consider the risk of appendiceal cancer. Some authors recommend routine interval appendectomy to rule out the possibility of malignancy rather than to avoid the risk of recurrence [7] [32] [33]. Recent retrospective studies report that the rate of appendiceal neoplasms in patients undergoing interval appendectomy is especially high in patients with complicated appendicitis ≥40 years of age [32] [33]. The rate is substantial, and surgeons should be aware of the risk of malignancy in patients with complicated appendicitis. However, a systematic review and meta-analysis showed a 7.4% incidence of recurrent appendicitis and a 1.2% incidence of malignant neoplasm in patients undergoing successful nonoperative management for complicated appendicitis. Based on these findings, the authors concluded that interval appendectomy is not necessary [4]. The role of appendectomy in complicated appendectomy for oncological reasons is debated. Investigating the cost-effectiveness of appendectomy in complicated appendectomy with a long-term follow-up regarding the possibility of malignancy in the appendix is an area requiring future research. Second, because of the lack of evidence on this subject, in the present study, the variables of the strategies were estimated mainly from meta-analysis using data over various periods. Therefore, with improvement in interventional radiology and increased use of laparoscopy, the relevance of those estimates may be questionable. However, probabilistic sensitivity analysis still found operative management to be cost effective in simulations. It is plausible that the finding of this study is generalizable in the wide range of values in our model. Third, few studies have evaluated quality of life in the early postoperative period after appendectomy. Therefore, we estimated utilities for the treatment strategies based on data related to other diseases and surgical procedures. If the utility of the procedures in patients with complicated appendicitis differed from our assumptions, our model outcomes could be compromised. Further studies are needed to better characterize the health states associated with the treatment of complicated appendicitis. Finally, our study did not provide sufficient data to assess minor and major complications individually. The impact on cost and utility is strongly influenced by the type of complication; therefore, it should be emphasized that substantial differences in cost and utility of complications could affect the model outcome.

## Conclusion

The study concluded that nonoperative management with ILA and nonoperative management without ILA were more costly and gained slight additional utility compared with operative management in the treatment of complicated appendicitis, at the set threshold. The results of the current study support cost-effectiveness of operative management with laparoscopic appendectomy in complicated appendicitis patients.

## Data Availability

The datasets used and/or analyzed during the current study available from the corresponding author on reasonable request.

## Abbreviations

ELA: Emergency laparoscopic appendectomy
ILA: Interval laparoscopic appendectomy
QALY: Quality-adjusted life years
HRQoL: Health-related quality life
ICER: Incremental cost-effectiveness ratio
WTP: Willingness-to-pay
